# Exploring Key Genes and Mechanisms in Respiratory Syncytial Virus-Infected BALB/c Mice *via* Multi-Organ Expression Profiles

**DOI:** 10.3389/fcimb.2022.858305

**Published:** 2022-05-02

**Authors:** Yu He, Zhili Wang, Jianhua Wei, Zhongying Yang, Luo Ren, Yu Deng, Shiyi Chen, Na Zang, Enmei Liu

**Affiliations:** Department of Respiratory Medicine Children’s Hospital of Chongqing Medical University, National Clinical Research Center for Child Health and Disorders, Ministry of Education Key Laboratory of Child Development and Disorders, Chongqing Key Laboratory of Pediatrics, Chongqing, China

**Keywords:** respiratory syncytial virus (RSV), multi-organ, hemopexin (Hpx), platelet degranulation, neutrophil degranulation

## Abstract

Respiratory syncytial virus (RSV) a leading cause of pediatric and adult morbidity and mortality worldwide. It can cause complications in multiple organs, thus increasing hospital stays and costs. However, RSV-based studies have primarily focused on effects in the lungs and blood, thereby potentially neglecting critical genes and pathways. Hence, studying RSV infection *via* a novel multi-organ approach is important. In this study, lung, intestine, brain, and spleen tissues from six BALB/c mice (6–8 weeks old; three in control group and three in RSV-infected group) were subjected to RNA sequencing. Differentially expressed genes (DEGs) in each organ were obtained and functional enrichment analysis was performed. We first used CIBERSORT to evaluate the immune-infiltration landscape. Subsequently, common DEGs (co-DEGs) among the four organs were analyzed to identify key genes and pathways. After quantitative reverse transcription-polymerase chain reaction, western blotting, and external validation analysis of key hub genes, their correlation with immune cells and potential functions were explored. We found that the host response to RSV infection varied among the four organs regarding gene expression profiles and immune cell infiltration. Analysis of the 16 co-DEGs indicated enrichment in the platelet and neutrophil degranulation pathways. Importantly, the key gene hemopexin (Hpx) was strongly correlated with the immune cell fraction in the lungs and may participate in the regulation of platelet activation and immune response.

## Introduction

Respiratory syncytial virus (RSV), an enveloped negative-strand RNA virus, is a member of the *Orthopneumovirus* genus within the Pneumoviridae family of the Mononegavirales order (Rima et al., 2017). First isolated from infants with lower respiratory illness in 1957 (Chanock et al., 1957), RSV remains a significant burden to global health and a leading cause of pediatric and adult morbidity and mortality worldwide (Falsey et al., 2005; Nair et al., 2010; Branche and Falsey, 2015). Evidence also indicates that RSV infection can cause complications in multiple organs, thus increasing disease burden (Willson et al., 2003).

Several factors have been identified as critical to the pathogenesis of RSV infection, including systemic innate immune response, inflammatory response, coagulation activation, T cell associated immune response, and viral factors, such as viral load and specific RSV genotype (Hasegawa et al., 2015; Heinonen et al., 2020; Dapat et al., 2021). However, most previous studies have concentrated on the effects of RSV on lungs and blood, and a comprehensive understanding of RSV pathogenesis from a more systematic perspective is lacking. Moreover, RSV-induced changes in multi-organ pathology may exhibit common mechanisms, which could help clarify the pathogenesis and potential therapeutic targets of RSV infection.

Complications caused by RSV infection have been observed and reported in many clinical studies. These complications can increase the risk of death and prolong hospital stays, and involve respiratory failure (Geoghegan et al., 2017; Kong et al., 2020), acute respiratory distress syndrome (Dahlem et al., 2007; Subissi et al., 2020), and pneumorrhagia (Soo and Inwald, 2017), as well as a spectrum of neurological complications, such as encephalitis, acute encephalopathy, complex seizures, hyponatremic seizures, and immune-mediated disorders with seizure (Saravanos et al., 2021). Digestive system complications, such as diarrhea (Liu et al., 2016), and hematological complications, such as secondary thrombocytosis and anemia (Zheng et al., 2016), have also been reported. At present, however, our understanding of the gene regulation and underlying mechanism of RSV-induced multi-organ damage remains poor. Heme-binding protein hemopexin (Hpx) is a 60-kDa plasma glycoprotein, which can not only clear heme but also acts as a multifunctional agent in important processes such as iron homeostasis, antioxidant protection, signaling pathways to promote cell survival and gene expression (Tolosano et al., 2010; Montecinos et al., 2019). Importantly, Hpx was reported to possess beneficial properties in lung and brain injury (Lam et al., 2016; Jung et al., 2017; Yang et al., 2018; Ashraf et al., 2020). Yet, the role of Hpx for RSV caused pathogenesis remains elusive.

In the present study, we aimed to clarify the effects of RSV infection on multiple organs in mice and to explore the key genes and mechanisms of RSV infection *via* multi-organ expression profiles. We performed deep transcriptomic analysis of lung, intestine, spleen, and brain tissues from RSV-infected female BALB/c mice. Our results demonstrated that Hpx and platelet and neutrophil degranulation played vital roles in multiple organs post-RSV infection, thus providing hints for potential therapies and biomarkers.

## Materials and Methods

### Ethics Statement

All experiments involving animals were carried out in accordance with the Guide for the Care and Use of Laboratory Animals and with approval from the Committee on the Ethics of Animal Experiments of Chongqing Medical University (Permit number: SYXK-(YU) 2012-0001). All surgeries were performed under anesthesia and every effort was made to minimize pain.

### Animals

Female BALB/c mice (6–8 weeks old) were obtained from the Experimental Animal Center of Chongqing Medical University (Chongqing, China) and maintained in a specific pathogen-free environment with a 12 h light-dark cycle at a humidity of 55% ± 5% and temperature of 25 ± 2°C. All mice were given free access to food and water.

### Infections

The RSV-A2 strain (VR-1540) was obtained from the American Type Culture Collection (ATCC, Manassas, VA, USA). The strain was grown in HEp-2 cells and purified by density gradient (Gias et al., 2008). The virus titer was 1.0 × 10^8^ PFU/ml, as determined by serial dilution plaque assay (McKimm-Breschkin, 2004). The BALB/c mice were infected with 60 μl of RSV-A2 *via* intranasal inoculation under 2%–5% isoflurane. Based on our previous study of lung tissue from RSV-infected mice, where antiviral interferon-α/β expression peaked at 12 h after infection (Ye et al., 2017), we chose the 12-h time point for sampling. At 12 h post-RSV infection, mice were anesthetized with CO_2_ and sacrificed by cervical dislocation. The lungs, brains, spleens, and colons (1–2 cm of colon near the ileocecal region) were immediately collected and frozen with liquid nitrogen for at least half an hour and then stored at -80°C.

### RNA Sequencing (RNA-Seq)

Lung, spleen, intestine, and brain samples from the control and RSV-infected groups (three mice each) were ground in liquid nitrogen and randomly sampled for total RNA extraction with TRIzol reagent (Invitrogen, CA, USA) following the manufacturer’s procedures. Total RNA quantity and purity were analyzed using a Bioanalyzer 2100 and RNA 6000 Nano LabChip Kit (Agilent, CA, USA) with an RNA integrity number (RIN) of >7.0. Poly (A) mRNA was isolated from 1 μg of total RNA using poly-T oligo magnetic beads (Invitrogen, CA, USA). Following purification, the mRNA was fragmented into 300-nt long oligonucleotides using divalent cations under elevated temperature. The cleaved mRNA fragments were then reverse transcribed to create a final cDNA library in accordance with the protocols of the mRNA Seq Sample Preparation Kit (Illumina, San Diego, USA) using the dUTP method. Average insert size for the paired-end libraries was 300 bp ( ± 50 bp). Paired-end sequencing (2 × 150 bp) on the Illumina NovaSeq™ 6000 platform was performed at LC-BIO Bio-Tech Ltd. (Hangzhou, China) following the vendor’s recommended protocols.

### Data Processing and DEG Screening

Raw counts were transformed by vst conversion and differentially expressed genes (DEGs) in the mouse groups were determined using the Bioconductor package “DESeq2” (version 1.26.0) (Anders and Huber, 2010). Statistical significance was set to |log2 fold-change (logFC)| > 1 and raw *p* < 0.05 for the spleen, intestine, and brain, but to |logFC| > 2 and raw *p* < 0.05 for the lung. Significance, fold-change, and gene expression patterns of the DEGs were visualized with volcano plots and heat maps using the OmicStudio tool (https://www.omicstudio.cn/tool). To identify common DEGs (co-DEGs) among the four organs, the online Venn tool (https://www.omicstudio.cn/tool/6) was used to draw a Venn diagram, and co-DEGs were retained for further analysis.

### Functional Annotation and Pathway Enrichment Analysis of DEGs and Co-DEGs

The biological functions of DEGs was identified by Gene Ontology (GO) (2017) and Kyoto Encyclopedia of Genes and Genomes (KEGG) (Kanehisa et al., 2008) pathway enrichment analyses using the R package “clusterProfiler” (Yu et al., 2012) (version 3.14.3). The GO and KEGG enrichment results were visualized using the OmicStudio tool (https://www.omicstudio.cn). Terms and pathways were considered significantly enriched at a cutoff of *p* < 0.05. For co-DEGs among the four organs, GO was used to investigate variations in biological functions and Metascape (http://metascape.org/gp/index.html) was used to investigate variations in pathways post-RSV infection.

### Gene Set Enrichment Analysis (GSEA)

To explore the potential function of key hub genes, samples were divided into high- and low-expression groups according to the median value of the key hub gene. The “clusterProfiler” package was used for GSEA based on gene expression profiles. Previously annotated gene sets “Mm.c2.cp.reactome.v7.1.entrez.gmt” and “Mm.c2.cp.kegg.v7.1.entrez.gmt” were chosen as the reference gene list. A default algorithm with 1 000 permutations was applied to calculate *p*-values and enrichment scores. The gene set size filters were minimum of 10, maximum of 1 000, *p* = 0.05, and pAdjustedMethod = “BH”. Cut-off criteria were set to | Normalized Enrichment Score (NES)| > 1 and *p* < 0.01.

### Immune Cell Infiltration in Organs Post-RSV Infection

The deconvolution algorithm CIBERSORT contains gene expression reference values from a signature matrix of 547 genes in 22 types of immune cells (Newman et al., 2015). To evaluate abundance of immune infiltrates in various organs, we uploaded the gene expression matrix data to the CIBERSORT (https://cibersort.stanford.edu/) web portal, and the algorithm was run using the LM22 signature for 100 permutations. The percentage of each immune cell type in the samples was calculated and displayed in a bar plot. The “vioplot” package (verison 0.3.7) (Hu, 2020) was then used to draw violin diagrams to visualize differences in immune cell infiltration. The Wilcoxon test was used to analyze differences in immune cell fractions between the RSV-infected mice and healthy controls.

### Hub Gene Selection and Analysis

A protein-protein interaction (PPI) network of DEGs was constructed using the Search Tool for the Retrieval of Interacting Genes/Proteins (STRING) (Snel et al., 2000) database. An interaction score > 0.4 was regarded as statistically significant. The PPI network was then visualized using Cytoscape (version 3.8.2) (Shannon et al., 2003) and the cytoHubba plug-in was employed to calculate the degree of interaction between DEGs, with the top five genes defined as hub genes.

### Validation of Hub Gene Expression

For gene validation, mRNA expression profiles in the lung tissues of RSV-infected mice were obtained from the Gene Expression Omnibus (GEO) database (accession number: GSE111861). GSE111861 contains three RSV-infected intact, three RSV-infected TLR4 mutant, and three wild-type mice. Preprocessed gene expression data and sample annotation were obtained from GEO (Series Matrix Files). Differential analysis was performed for GSE111861 using the “limma” package (version 3.42.2). A boxplot was generated to visualize the gene expression data using the “ggplot2” package (version 3.3.4) (Ginestet, 2011).

### RNA Extraction and Quantitative Reverse Transcription Polymerase Chain Reaction (qRT-PCR)

Non-infected and 12, 24, and 48 h post-RSV infected mice were sacrificed and whole lungs, intestines, brains, and spleens were removed. Total RNA was extracted from the tissues using TRIzol Reagent (Invitrogen, USA) and an RNA Extraction Kit (TR205, Tianmo Biotech, China). Total RNA concentration and purity were assessed using a NanoDrop 2000, and 1 μg of RNA was used for first-strand cDNA synthesis with a PrimeScript RT Kit (TaKaRa, Japan) according to the manufacturer’s instructions. The cDNA was amplified using a VeriQuest Fast SYBR Green qPCR Kit (Invitrogen, USA) with the following primers: *Hpx* (forward 5’TAGCTGGCCCATTGCTCATC3’; reverse 5’-GAGGGCTCCCAAGTTCCTTC-3’) and *Gapdh* (forward 5’-CATCACTGCCACCCAGAAGACTG-3’; reverse 5’-ATGCCAGTGAGCTTCCCGTTCAG-3’). The PCR cycle conditions were: 95°C for 2 min, then 40 cycles at 95°C for 5 s and 60 °C for 30 s. The fold-change was obtained using the 2^-ΔΔCt^ method with *Gapdh* calibration. RSV N gene were performed using primers and probes: forward primer, 5’-AGATCAACTTCTGTCATCCAGCAA -3’, reverse primer, 5’-TTCTGCACATCATAATTAGGAGTATCAAT-3’ and probe, 5’-FAM-CACCATCCAACGGAGCACAGGAGAT-BHQ1-3’. The PCR cycle conditions were: 50°C for 2 min, 95°C for 10 min, then 40 cycles at 95°C for 15 s and 60°C for 1 min. For absolute quantification, the RSV N gene copy was calculated using a plasmid DNA standard curve.

### Protein Extraction and Western Blot Analysis

Total protein in tissues was extracted using a Total Protein Extraction Kit (KGP2100, KeyGEN, China). Western blotting was performed following previously reported procedures (Ye et al., 2017). The primary antibody was anti Hpx (ab133415, Abcam, USA). Gapdh (200306, ZENBIO, China) was used as a loading control. Image collection was performed using Quantity One v4.6.2 (USA) and densitometry analysis was conducted using ImageJ v2.0.0 (USA).

### Analysis of Correlations Between Key Hub Genes and Immune Cells

Pearson correlation analysis of key hub genes and immune cells was performed to analyze the immune mechanism during RSV infection using the “ggstatsplot” R package (Patil, 2021).

### Statistical Analysis

All quantitative data are presented as mean ± standard error of the mean (SEM). The proportion of innate immune cells between groups was compared using the unpaired Student’s *t*-test. Pearson correlation analysis was performed to reveal the relationship between key hub genes and immune cells. Differences in mRNA expression between/among groups were determined using Student’s *t*-test or one-way analysis of variance (ANOVA) respectively followed by Tukey’s multiple comparisons in GraphPad Prism v7.0.4 (GraphPad Software Inc., USA). A *p*-value of less than 0.05 was considered statistically significant.

## Results

### DEG Identification in Four Organs of RSV-Infected Mice

**Figure 1:**
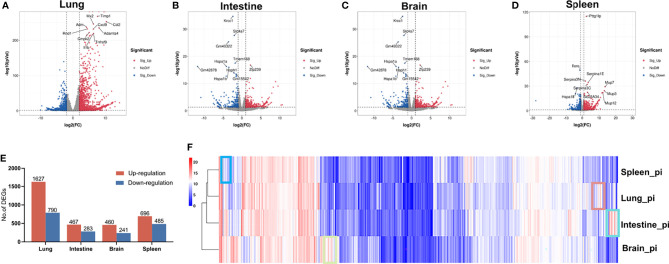
Gene expression patterns in lung, intestine, brain, and spleen of control and RSV-infected mice. **(A–D)** Volcano plots of DEGs in lung, intestine, brain, and spleen of control and RSV-infected mice. **(E)** Distribution of up- and down-regulated DEGs in lung, intestine, brain, and spleen. **(F)** Heatmap of gene expression levels in organs after hierarchical clustering. Selected boxes show organ-specific expressed genes.

The distribution of DEGs in the different organs is shown in the volcano plot in **Figures 1A–D**, with the 10 most significant DEGs marked. In total, we identified 2 417, 750, 701, and 1181 DEGs in the lung, intestine, brain, and spleen, respectively (**Figure 1E**). For more accurate functional enrichment, the DEG threshold for the lung was set to |logFC| > 2. Among the tissue types, the lungs harbored the greatest number of DEGs, indicating that the lungs exhibited the most significant mRNA changes in response to RSV. The significantly up- and down-regulated DEGs are displayed in the heat map (**Figure S1**), which shows different distributions of gene expression patterns between the RSV-infected and control mice. Gene expression in the four organs post-RSV infection, after hierarchical clustering, is shown in the heatmap in **Figure 1F**, with genes in boxes indicating tissue-specific gene expression signatures. Clustering analysis revealed that the lung and spleen had the most similar gene expression profiles post-RSV infection (**Figure 1F**).

### Functional Enrichment Analysis of DEGs in Four Organs of RSV-Infected Mice

**Figure 2:**
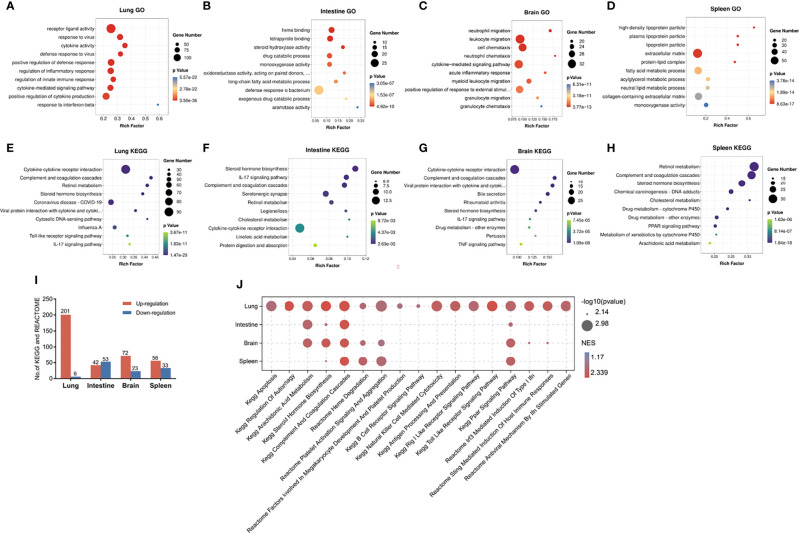
Enrichment analysis of DEGs in lung, intestine, brain, and spleen of RSV-infected mice. **(A–D)** Top 10 enriched GO terms in DEGs in lung, intestine, brain, and spleen, respectively. **(E–H)** Top 10 enriched KEGG pathways in DEGs in lung, intestine, brain, and spleen, respectively. An adjusted *p-*value < 0.05 was considered significant in GO and KEGG analysis. **(I)** Distribution of significantly up- and down-regulated KEGG and Reactome pathways detected by GSEA in four organs (*p* < 0.05). **(J)** Visualization of up-regulated pathways in four organs using OmicShare tools (https://www.omicshare.com/tools). GO, Gene Ontology; KEGG, Kyoto Encyclopedia of Genes and Genomes.

GO and KEGG enrichment analyses were used to evaluate the potential functions of the DEGs in the lung, intestine, brain, and spleen of RSV-infected mice. **Figures 2A–H** show the top 10 enriched GO and KEGG terms in the four organs. Based on GO analysis, the DEGs in the lung were mainly enriched in virus defense response, cytokine, and innate immune response (**Figure 2A**). For KEGG analysis, the lung DEGs were mainly enriched in the cytokine-cytokine receptor interaction, completement and coagulation cascades and retinol metabolism pathways (**Figure 2E**). In the intestine, DEGs were significantly enriched in heme binding, tetrapyrrole binding, steroid hydroxylase activity, and long-chain fatty acid metabolic process based on GO analysis (**Figure 2B**), and enriched in the steroid hormone biosynthesis, completement and coagulation, and IL-17 pathways based on KEGG analysis (**Figure 2F**). In the brain DEGs, enriched GO terms included neutrophil and leukocyte migration and cell and neutrophil chemotaxis (**Figure 2C**) and enriched KEGG pathways included the completement and coagulation cascade and IL-17 signaling pathways, as found in the lung, as well as the cytokine-cytokine receptor interaction and tumor necrosis factor (TNF) signaling pathways (**Figure 2G**). In the spleen DEGs, enriched GO terms included high density lipoprotein particle and steroid metabolic process (**Figure 2D**) and enriched KEGG pathways included the steroid hormone biosynthesis and completement and coagulation signaling pathways (**Figure 2H**). These results indicate that the different organs showed both divergent and common responses to RSV infection.

Due to the limitations of DEG enrichment analysis, key genes with moderate expression changes may be omitted by DEG criteria and important pathways underlying pathophysiological changes may be overlooked. Thus, we conducted GSEA to further investigate functional changes correlated with RSV infection in different organs. Based on GSEA, we identified 201, 42, 72, and 56 markedly up-regulated pathways (**Figure 2I**), as well as six, 53, 23, and 33 down-regulated pathways in the lung, intestine, brain, and spleen, respectively (**Figure 2I**). Up-regulated processes in the lung mainly involved immune- and virus recognition-related pathways, which were not significantly increased in the other three organs (**Figure 2J**). Moreover, the apoptosis and regulation of autophagy pathways were only up-regulated in the lung. Interestingly, the lung and brain shared several elevated pathways, including interferon- and STING-mediated induction of host immune responses, thus suggesting a relationship between the lung and brain in RSV infection.

Although all the four organs had immune response changes post RSV infection, we wondered if the immune responses observed in these organs were secondary to lung as part of a systemic response or were the intestine, brain and spleen directly infected by RSV. Therefore, we examined RSV N gene copies in the four organs, and found that RSV genome was detected only in lung (**Figure S2**), indicating RSV can’t directly infect intestine, brain and spleen. Together, these findings suggest that the lungs are a major virus-host battlefield during RSV infection and the intestines, brains and spleens could be influenced secondary to lung.

### Immune Cell Profile Analysis of Four Organs in RSV-Infected Mice

**Figure 3:**
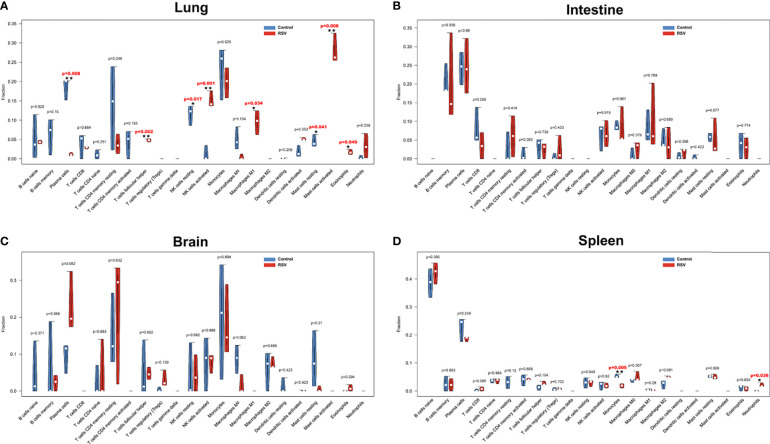
Violin diagram of proportion of 22 types of immune cells in four organs. **(A)** Lung, **(B)** intestine, **(C)** brain, and **(D)** spleen showed differences in immune cell infiltration between RSV-infected and control mice at 12 h post-RSV infection. *p < 0.05, **p < 0.01, compared with control group respectively.

To further assess immune response changes in the four organs post RSV infection, we conducted immune cell infiltration with CIBERSORT. The percentages of the 22 types of immune cells in the four organs are shown in the violin plots (**Figure 3**). Compared with the control group, plasma cells, resting natural killer (NK) cells, M0 macrophages, and resting mast cells were decreased in the lungs of RSV-infected mice, whereas T follicular helper (TFH) cells, activated NK cells, M1 macrophages, activated mast cells, and eosinophils were increased (**Figure 3A**). Several other studies have also reported increased TFH cells, activated NK cells, and eosinophils in the lung during RSV infection (Harrison et al., 1999; Li et al., 2012; Pyle et al., 2021). These findings demonstrate the reliability of CIBERSORT in predicting immune cell infiltration. Interestingly, increased M1 macrophages and activated mast cells and decreased resting mast cells have not been reported previously in the lung post-RSV infection. Our results also showed that immune cells were not significantly altered in the intestine or brain (**Figures 3B, C**), but monocytes were decreased and neutrophils were increased in the spleen, respectively (**Figure 3D**). These changes in neutrophils and monocytes in the spleen post-RSV infection have not been reported previously. Our study demonstrated the occurrence of distinct immune cell populations among different organs during infection; however, the specific mechanism and function of some types remain to be elucidated.

### Identification of Key Genes and Pathways in RSV-Infected Mice in Four Organs

**Figure 4:**
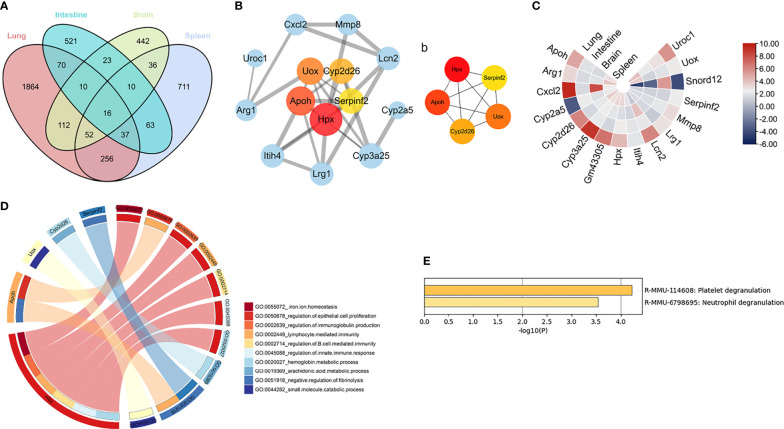
Key genes and pathways in four organs **(A)** Venn diagram showing overlapping DEGs in four organs. Four organs shared 16 overlapping co-DEGs. **(B)** PPI network of co-DEGs; nodes in pink to red denote five hub genes identified by “cytoHubba”. Thicker edges indicate stronger interactions between two proteins. Larger protein nodes indicate higher number of protein nodes with which the interaction is formed. (b) Networks of the five hub genes identified by “cytoHubba”. Pink to red scale denotes *p*-value calculated by MCC method. **(C)** Heatmap of co-DEGs derived from integrated analysis. Each circle represents one organ, each sector represents one gene, and gradual color change from blue to red represents changing up-regulation. **(D)** GO term enrichment analysis of co-DEGs. Circos plot displays significantly enriched GO terms for five hub genes. **(E)** All enriched terms were based on comprehensive enrichment analysis of KEGG and Reactome databases with “Metascape”. Co-DEGs, common differentially expressed genes.

To elucidate the common mechanism underlying multi-organ pathology after RSV infection, co-DEGs were obtained for further analysis. As shown in **Figure 4A**, 16 genes overlapped in the four organs. Subsequently, the PPI networks of these co-DEGs were constructed and 5 hub genes were screened, i.e., Hpx, Apoh, Uox, Cyp2d26 and Serpinf2, with Hpx identified as the most key hub gene by cytoHubba (**Figure 4B**). Expression of the five hub genes in the different organs was shown in **Figure 4C** and GO analysis of the hub genes was shown in **Figure 4D**. According to Metascape pathway enrichment analysis, the 16 co-DEGs were enriched in the platelet degranulation and neutrophil degranulation pathways (**Figure 4E**). These results suggest that aberrantly expressed Hpx and activation of neutrophil degranulation and neutrophil degranulation pathways may play crucial role in the RSV induced multi-organ pathology. As Hpx was identified as the most key hub gene in the PPI network, we chose it for further analysis.

### Biological and External Validation of Hpx Expression

**Figure 5:**
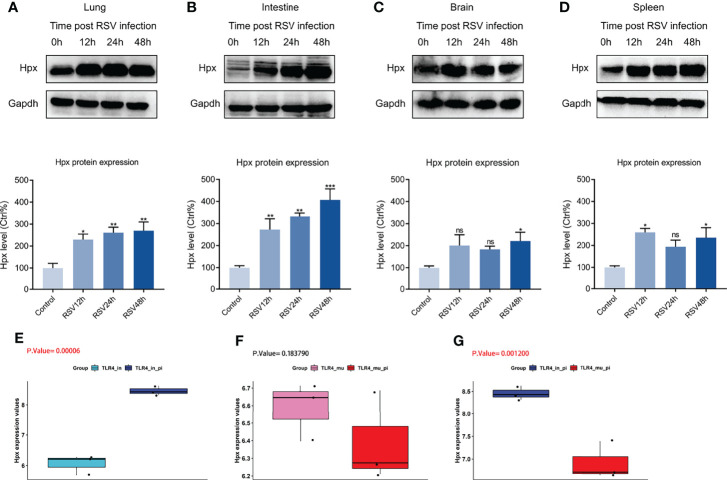
Western blotting and GEO database validation of Hpx expression in four organs post-RSV infection. **(A–D)** Western blotting of Hpx protein levels in RSV-infected BALB/c mice at different time points in lung, intestine, brain, and spleen. *p* < 0.05. *p < 0.05, **p < 0.01, ***p < 0.001, compared with control group. Hpx expression in **(E)** TLR4-intact and TLR4-intact infected mice, **(F)** TLR4 mutant and TLR4 mutant infected mice, **(G)** TLR4 intact infected and TLR4 mutant infected mice based on GEO database. ns. no.

We constructed RSV-infection mouse models at 12, 24, and 48 h post-RSV infection to confirm the RNA-Seq results. Western blotting showed that the protein expression level of Hpx was elevated in the four organs post-RSV infection up to 48 h (**Figures 5A–D**), as confirmed by qRT-PCR (**Figure S3**). To further validate our results, we downloaded the GSE111861 dataset from the GEO database and analyzed the differential expression of Hpx mRNA post-RSV infection in the lung. By comparing the expression levels of Hpx in four groups of the GSE111861 dataset (i.e., TLR4 intact, TLR4 mutant, TLR4 intact post-RSV infection, and TLR4 mutant post-RSV infection), we found that Hpx expression was elevated in TLR4 intact mice at day 1 post-RSV infection, similar to our data (**Figure 5E**). Interestingly, Hpx expression was not elevated in TLR4 mutant mice post-RSV infection (**Figure 5F**). Therefore, Hpx expression in TLR4 intact mice post-RSV infection was significantly increased compared to the TLR4 mutant groups post-RSV infection (**Figure 5G**). Notably, in the research based on GSE111861 dataset, RSV-infected mice with lower Hpx expression (TLR4 mutant groups post-RSV infection) had lower virus titer and lighter lung inflammation, indicating that Hpx expression may associate with severity of RSV infection.

### Correlation Analysis of Hpx With Immune Cells and Predicted Functions of Hpx

**Figure 6:**
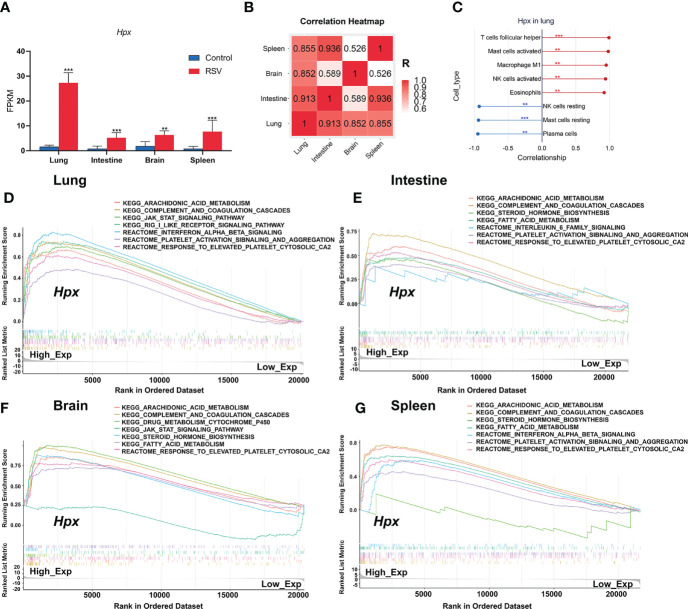
Correlation between immune cells and Hpx expression and pathways associated with Hpx alteration predicted by GSEA. **(A)** Hpx expression in transcriptome results. **(B)** Hpx expression correlation heatmap in four organs. **(C)** Scatter diagrams from correlation analysis in lung. Y-axis represents Hpx, x-axis represents immune cell content, as defined by CIBERSORT algorithm. **(D)** Predicted pathways in lung, **(E)** intestine, **(F)** brain, **(G)** and spleen. Comparative changes between high- and low-expression groups. All terms shown are significantly enriched at NES> 1 and *p* < 0.05. **p < 0.01, ***p < 0.001, number of asterisks represent degree of importance.

To assess if Hpx expression in the four organs correlate with each other, we visualized the Hpx expression changes post RSV infection in the four organs in one histogram and then showed the correlation of Hpx expression among the four organs with a correlation heat map of fragments per kilobase million (FPKM) values. Results showed the Hpx was significantly increased in all the four organs with the lung had the maximum fold change of Hpx mRNA expression (**Figure 6A**). Hpx mRNA expression levels in lung, intestine, and spleen were significantly positively correlated with each other and correlations of Hpx expression between intestine, spleen and brain were much lower, though it was high between lung and brain (**Figure 6B**).

To evaluate the relationship between Hpx expression and immune cell infiltration status, we further used Pearson correlation analysis to determine the correlations between Hpx expression and immune cell fractions. Correlation analysis between Hpx and immune cell fractions in the lung showed that Hpx was strongly positively correlated with increased TFH cells, activated mast cells, M1 macrophages, activated NK cells, and eosinophils, and negatively correlated with decreased plasma cells, resting NK cells, and resting mast cells (**Figure 6C**). In the spleen, Hpx showed positive association with neutrophils and negative association trends with monocytes (**Figure S4**).

Finally, to explore the potential function of Hpx post-RSV infection, we conducted GSEA to analyze enriched KEGG and Reactome pathways based on Hpx expression in the four organs. Gene sets associated with the completement and coagulation cascades, JAK-STAT, interferon-α/β, platelet activation, and aggregation signaling pathways were highly up-regulated in the groups with high Hpx expression (**Figures 6D–G**), indicating that Hpx may act together with these pathways. Together, these results suggest that Hpx may be vital in the multi-organ pathogenesis of RSV infection for its potential participation in immune cell infiltration and pathways.

## Discussion

In this study, we applied multi-organ system analysis and combined transcriptional profiling and experimental validation to gain insight into the key genes and mechanisms associated with RSV infection. Based on bioinformatics analysis of DEGs and enriched pathways, RSV infection caused significant host responses in multiple organs. Immune cell infiltration analysis showed significant changes in immune cells in the lung and spleen post-RSV infection, which may contribute to RSV pathology. Furthermore, enrichment analysis of the 16 co-DEGs shared by the four studied organs revealed that platelet and neutrophil degranulation may be involved in the multi-organ features of RSV pathogenesis. In addition, Hpx was identified as a critical gene by CytoHubba prediction and biological and external validation. Notably, Hpx was strongly correlated with increased immune cell fractions in the lung and significantly associated with immune and platelet activation pathways.

Results showed that gene expression changes and enriched biological processes and pathways post-RSV infection were organ specific. The lung harbored the greatest number of DEGs and shared the most DEGs with the spleen. The lung and spleen also showed the most similar expression profiles post-RSV infection. In addition, virus and interferon-α/β response, inflammatory and cytokine response, and innate immune response were enriched in the lungs, but not in the other three organs. Interestingly, neutrophil and leukocyte migration and cell and neutrophil chemotaxis processes were significantly enriched in the brain. Neutrophils can cross the blood-brain barrier (BBB) and are among the first circulating leukocytes to arrive at the ischemic hemisphere (Enzmann et al., 2013). It is reported that neutrophil depletion attenuates infarction size in mouse models (Herz et al., 2015) and inhibition of neutrophil migration reduces brain injury and behavioral impairment (Neumann et al., 2015). Clinically, diffusion-weighted magnetic resonance imaging (DWI) show that greater migration of neutrophils to the ischemic hemisphere can result in larger infarct volumes in the acute phase after stroke (Buck et al., 2008). As RSV infection can cause neurological consequences in patients, such as communicative disabilities and seizure (Peña et al., 2020; Saravanos et al., 2021), our results indicate that neutrophils may play a vital role in the neurological complications caused by RSV infection. Notably, the “factors involved in megakaryocyte development and platelet production” pathway was specifically enriched in the lung, which may explain platelet elevation post-RSV infection (Zheng et al., 2016).

In addition, for the first time, we explored infiltration of the 22 immune cell types in multiple organs post-RSV infection. Overall, RSV infection primarily affected immune cell infiltration in the lung and spleen, rather than the intestine and brain. This may be because altered innate immune cells in the lung contribute to host defense against RSV infection but may also cause excessive damage. In addition, as the spleen is the largest peripheral immune organ in the body, changes in splenic immune cell populations when infection occurs may also play a vital role in anti-infection immunity. To the best of our knowledge, our study is the first to report on decreased resting mast cells and M0 macrophages and increased activated mast cells and M1 macrophages in the lung post-RSV infection. Although previous research has reported that RSV can induce mast cell degranulation (Dakhama et al., 2004), its effects on mast cell quantity remain unknown. Interestingly, one research reported that numbers of bronchiolar mast cells increased post Sendai virus inoculation, and the increase was associated with airway hyperresponsiveness and heightened allergic airway inflammatory reactions (Castleman et al., 1990). Another research revealed lung mast cells transiently accumulated post influenza infection and might contribute to virus-induced exacerbation of allergic airway inflammation such as asthma (Zarnegar et al., 2017). RSV increases susceptibility to allergic asthma (Krishnamoorthy et al., 2012) and is one of the most important triggers of asthma exacerbations (Jartti et al., 2020). However, the roles of mast cell in RSV-associated allergic asthma and RSV-related asthma exacerbations have yet to be explored, and thus studies on the functionality of increased infiltration of mast cells after RSV infection are warranted. Our results also showed a decrease in M0 macrophages and increase in M1 macrophages at 12 h post-RSV infection, indicating that M0 macrophages may differentiate into M1 macrophages after infection. Additionally, murine monocytes are recruited to the lung after RSV infection where they differentiate into macrophages (Goritzka et al., 2015), which may also lead to the increase in the percentage of M1 macrophages. In the exudative phase of acute lung injury/acute respiratory distress syndrome, lung M1 polarized macrophages increase, which can release TNF-α, interleukin 1 (IL-1), nitric oxide, and reactive oxygen species, thus inducing a severe inflammatory response (Patel et al., 2017). Similarly, the increase in M1 macrophages reported in the current study may play a key role in the early stages of lung inflammation post-RSV infection. We also detected a decrease in monocytes and increase in neutrophils in the spleen post RSV infection, which has not been reported previously. Research has shown that expanded lung interstitial macrophages are partly mobilized from spleen monocytes after exposure to bacterial CpG DNA (Sabatel et al., 2017). However, whether RSV infection also mobilizes splenic monocytes into lung macrophages is not clear. At least two populations of neutrophils have been shown in human spleens; however, these populations were induced postnatally by local signals (e.g., IL-10 and granulocyte-macrophage colony stimulating factor (GM-CSF)) and participated in antimicrobial immunoglobulin production (Puga et al., 2011; Chorny et al., 2016). As shown in **Figure 3D**, the fraction of neutrophils was almost zero before infection, but this increased to 2% post-RSV infection. However, the role of increased neutrophils in the spleen remains unknown and requires further investigation. Our results on immune cell infiltration in the four organs provide a map of immune cell changes of the four organs and highlight several immune cell subgroups not previously reported in RSV infection.

Hpx was identified as a key gene *via* multi-organ integration analysis. Notably, Hpx expression remained elevated up to 48 h post-RSV infection (**Figures 5A–D**). Wang et al. also reported that Hpx expression is significantly elevated in the rat lung, even 7 days post-RSV infection (Wang et al., 2016), suggesting that Hpx may be consistently elevated in early RSV infection. Moreover, we found that Hpx was strongly correlated with infiltrating immune cells in the lung and associated with several key pathways, such as platelet activation and aggregation signaling and interferon-α/β signaling. As a 60-kDa plasma glycoprotein composed of a single 439-amino acid peptide chain, Hpx binds heme with high affinity to protect against hemoglobin-derived heme toxicity and mitigate heme-mediated effects on immune, endothelial, and stem cells that collectively drive inflammation and perturb vascular hemostasis and BBB function (Montecinos et al., 2019). In the lung, Hpx can down-regulate proinflammatory cytokine production, reduce acute lung injury, and increase survival rates in a mouse model of endotoxemia (Jung et al., 2017), and can improve lung function and survival in a mouse model of bromine gas inhalation (Lam et al., 2016). In the brain, Hpx can protect synaptic plasticity and BBB integrity from heme toxicity (Yang et al., 2018) and higher Hpx levels are correlated with better hippocampal metabolism and cognitive performance (Ashraf et al., 2020). These studies indicate that elevated Hpx may be protective in multiple organs post-RSV infection. Counterintuitively, we analyzed the GSE111861 dataset on day 1 post-RSV infection and found that Hpx was decreased in the lungs of RSV-infected TLR4 mutant mice, which showed milder lung pathology and lower viral titers (Marzec et al., 2019), compared to RSV-infected TLR4 intact mice. Takagi et al. reported that Hpx may participate in the small intestine damage induced by non-steroidal anti-inflammatory drugs (Takagi et al., 2012). Higher serum levels of Hpx are also implicated in serious dengue fever (Ray et al., 2012). Hence, we suspect that Hpx may be a therapy target, as well as a biomarker that can predict host response and disease severity in RSV infection.

Platelet degranulation and neutrophil degranulation were identified as key pathways *via* multi-organ transcriptomic analysis of the 16 co-DEGs shared by the four organs. Neutrophil degranulation can induce the release of chemokines (IL-8, MIP-1α, MIP-1β) and granule enzymes, which may cause pulmonary pathology in RSV bronchiolitis (Jaovisidha et al., 1999). Neutrophils can result in both airway damage and viral clearance (Deng et al., 2020). Notably, a large number of neutrophils are recruited into the airways of children with severe RSV disease (McNamara et al., 2003) and overactivation of neutrophils is a blood transcriptional signature of severe RSV infection (Heinonen et al., 2020). However, Kirsebom et al. reported that neutrophils didn’t impact viral load and disease severity in RSV infection (Kirsebom et al.). Notably, they used C57BL/6 mice instead of BALB/c mice and assessed disease severity with weight loss rather than lung pathology. The differences in experimental design may account for these divergent results. Our results, combined with most previous studies, suggest that neutrophil degranulation may play a vital role in RSV multi-organ damage. As for platelet degranulation, few studies have examined its role in RSV infection. Platelet degranulation is an important marker of platelet activation. Compared with girls, boys were reported to have a higher incidence of bronchiolitis with higher activated platelets (De Jacobis et al., 2020). In addition, genes related to platelets were up-regulated in children with severe RSV infection (Dapat et al., 2021). In COVID-19 patients, platelet degranulation may serve as a biomarker for disease severity (Shen et al., 2020) and platelet degranulation dysfunction was closely related with illness severity (Shu et al., 2020). However, whether neutrophil and platelet degranulation can predict the severity of RSV infection and multi-organ damage needs to be further investigated.

Understanding the host response *via* multi-organ transcriptomic study has several strengths. By constructing multi-organ gene expression profiles, we identified key genes and pathways that may have been overlooked in studies on a single organ. Additionally, by constructing a multi-organ genome map, we discovered several connections and differences among organs post-RSV infection in terms of gene expression patterns and immune cell infiltration. These findings may help clarify host responses and multi-organ complications caused by RSV.

This study also has several limitations. Our transcriptome data provided a snapshot of gene expression at a single representative time point based on our previous research on lungs post-RSV infection (Ye et al., 2017), which may miss time points when significant changes occurred in other organs. Thus, we validated continuous Hpx expression levels after RSV infection at multiple time points in multiple organs. Another limitation is that we did not validate the mechanisms explored by this study due to a lack of mouse model of severe RSV infection. Thus, the specific molecular mechanism underlying RSV infection warrants further exploration. Constructing Hpx knockout mice to explore the regulatory effects of Hpx on multiple organs during RSV infection will be the focus of our next study.

In summary, this multi-organ transcriptomic study reveals that RSV infection causes significant host responses in multiple organs and sheds light on the vital role of Hpx and platelet and neutrophil degranulation in RSV infection, which may contribute to multi-organ pathologic changes. By constructing a multi-organ genome map, we gain comprehensive understanding of RSV-associated host responses, as well as provide hints of potential therapy and biomarker to predict host responses.

## Data Availability Statement

The datasets presented in this study can be found in online repositories. The names of the repository/repositories and accession number(s) can be found below: GEO database, accession number GSE197868.

## Ethics Statement

The animal study was reviewed and approved by Committee on the Ethics of Animal Experiments of Chongqing Medical University.

## Author Contributions

YH, NZ, and EL designed the study. YH, ZW, JW, and ZY analyzed data. YH performed the experiments and wrote the manuscript. LR, YD, SC, NZ, and EL edited and reviewed the manuscript. All authors contributed to the article and approved the submitted version.

## Conflict of Interest

The authors declare that the research was conducted in the absence of any commercial or financial relationships that could be construed as a potential conflict of interest.

## Publisher’s Note

All claims expressed in this article are solely those of the authors and do not necessarily represent those of their affiliated organizations, or those of the publisher, the editors and the reviewers. Any product that may be evaluated in this article, or claim that may be made by its manufacturer, is not guaranteed or endorsed by the publisher.
