# Supplementary material for: Exploring Key Genes and Mechanisms in Respiratory Syncytial Virus-Infected BALB/c Mice via Multi-Organ Expression Profiles
Source: Front Cell Infect Microbiol. 2022 May 2;12:858305. doi: 10.3389/fcimb.2022.858305 (PMC9109604; doi:10.3389/fcimb.2022.858305)

Figure S1. Heat map showing expression pattern between control and RSV-infected BALB/c mice in (A) lung, (B) intestine, (C) brain, (D) and spleen, respectively.

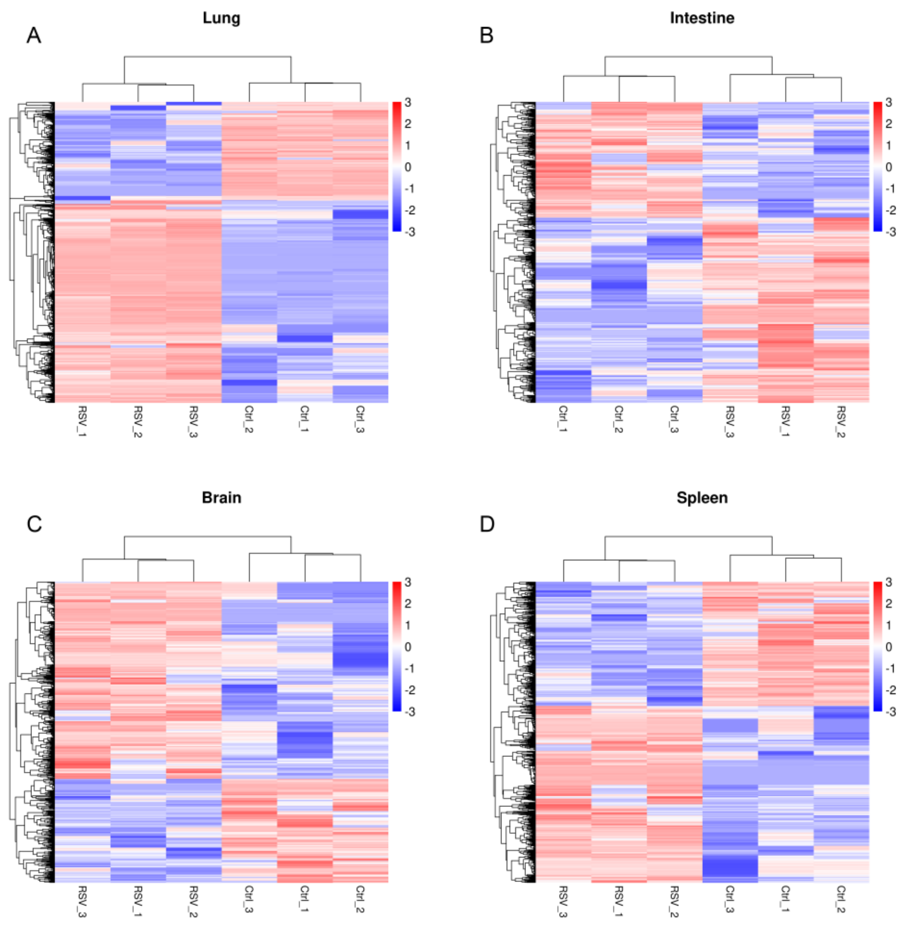

Supplement: Supplementary file 1 [file DataSheet_1.pdf]
