# Supplementary figures and images for: Exploring Key Genes and Mechanisms in Respiratory Syncytial Virus-Infected BALB/c Mice via Multi-Organ Expression Profiles
Source: Front Cell Infect Microbiol. 2022 May 2;12:858305. doi: 10.3389/fcimb.2022.858305 (PMC9109604; doi:10.3389/fcimb.2022.858305)

**Figure S2.** RSV N gene copies in lung, intestine, brain and spleen at 12h post RSV infection.

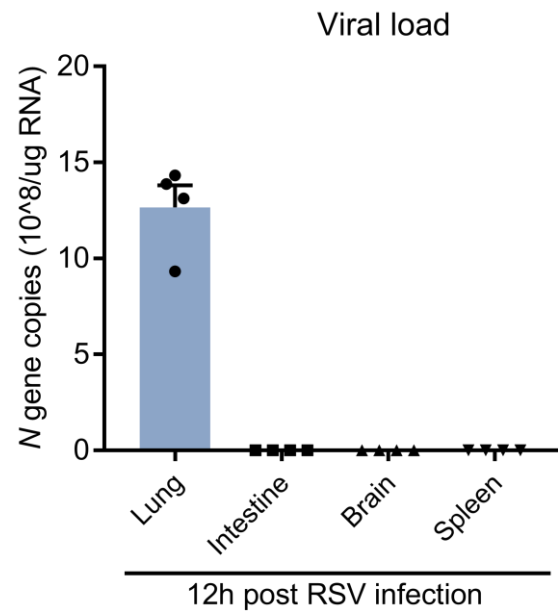

Supplement: Supplementary file 2 [file DataSheet_2.pdf]
