# Supplementary material for: Exploring Key Genes and Mechanisms in Respiratory Syncytial Virus-Infected BALB/c Mice via Multi-Organ Expression Profiles
Source: Front Cell Infect Microbiol. 2022 May 2;12:858305. doi: 10.3389/fcimb.2022.858305 (PMC9109604; doi:10.3389/fcimb.2022.858305)

Figure S3. qRT-PCR validation of Hpx expression in control and RSV-infected BALB/c mice in (**A**) lung, (**B**) intestine, (**C**) brain, and (**D**) spleen, respectively. **p* < 0.05, ****p* < 0.001, compared with control group.

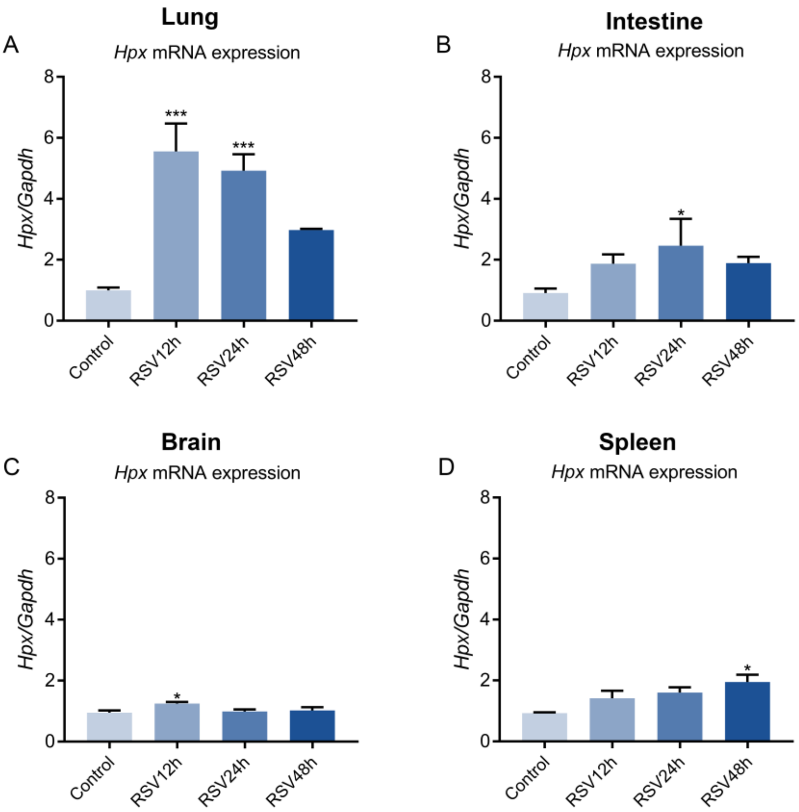

Supplement: Supplementary file 3 [file DataSheet_3.pdf]
