# Supplementary material for: Exploring Key Genes and Mechanisms in Respiratory Syncytial Virus-Infected BALB/c Mice via Multi-Organ Expression Profiles
Source: Front Cell Infect Microbiol. 2022 May 2;12:858305. doi: 10.3389/fcimb.2022.858305 (PMC9109604; doi:10.3389/fcimb.2022.858305)

Figure S4. Scatter diagram of correlation analysis in spleen. Y-axis represents Hpx, x-axis represents immune cell content, as defined by CIBERSORT algorithm. *p < 0.05, number of asterisks represent degree of importance.

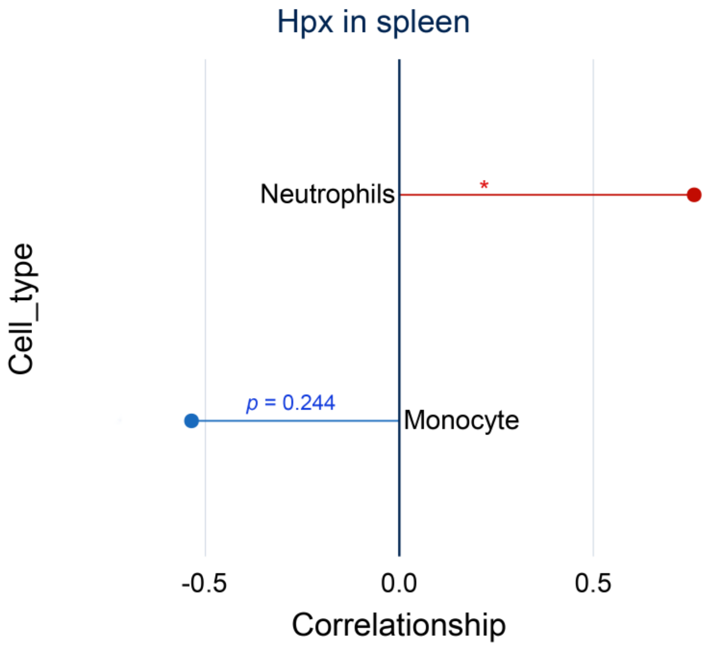

Supplement: Supplementary file 4 [file DataSheet_4.pdf]
